# Some Thoughts on AI Stimulated by Michael Wooldridge’s Book “The Road to Conscious Machines. The Story of AI”

**DOI:** 10.1007/s13218-022-00769-3

**Published:** 2022-07-28

**Authors:** W. F. Bodmer

**Affiliations:** Department of Oncology and Weatherall Institue of Molecular Medicine, University of Oxford, Oxford, UK

## Abstract

Following a brief over view of the contents of Michael Wooldridge's book I give an account of my own background in computing and AI. I then cover a range of topics stimulated by reading the book including machine learning's relationship to AI, applications to medical areas, the need to consider probabilistic effects on decisions, the importance of self-reproduction and whether AI can be made moral. I finish with a discussion of the mind–brain relationship and what makes us human.

## Introduction

Michael Wooldridge has written a stimulating book on AI that should be especially valuable for the general reader but also of interest to AI experts. It is not a textbook in any conventional sense. It starts with a well presented history of AI with its ups and downs, including a discussion of Turing’s key contributions and a description of virtual machines. The challenges faced by computing and the development of AI are well illustrated by Fig. 1 in Wooldrigde's book. This lists the potential tasks in order of difficulty from simply performing arithmetic to playing complex games, the development of driverless cars and finally to ‘Human-level general intelligence’ which is very far from being solved and may never be. AI has undoubtedly led to some extraordinary advances in applications, for example, to face recognition, automated acceptable language translation and voice recognition, valuable diagnostic assistance in medical areas such as the interpretation of mammograms for the early detection of breast cancer and, most recently, unravelling the rules by which proteins, the complex major functioning molecules of life, take up their natural shapes. Michael Wooldridge clearly shows, however, that even using computers for the apparently simple task of moving an object from one place to another, a foretaste of real-world robots, is far from trivial. Driverless cars will involve stepwise development and different levels of implementation from basic cruise control to the not by any means yet achieved, ‘no steering wheel’. However good the technology, serious accidents are bound to occur, and it should always be remembered that there is no such thing as ‘bug free’ software. Will adaptation of vehicle management, including special roads, be acceptable and affordable within the foreseeable future? There are already significant concerns about ‘algorithmic bias’ with respect to ethnic origins and gender in, for example, face recognition and disease diagnosis programmes. Will people be willing to accept AI for help in deciding who is guilty of a crime, with no intervention of a real living person? These are just a few examples of the problems discussed, especially in the latter part of the book, and well represented by the topics list in a useful and interesting glossary.

## My Computing and AI Background

I thought it might help to relate some of my more detailed comments on issues raised in Michael Wooldridge’s book to my own background in computing and AI.

Having been taken as a schoolboy in Manchester to see the original Williams computer, my first programming was on the Ferranti Mark1*commercial version of this computer when, on a summer job in 1956, I was calculating heat transfer coefficients for a plane that never flew. That was arithmetic at its most basic and yet it seemed like a bit of magic at the time. Little did I then realise what a huge influence that early experience of computing would have on my future career. Just about a year later, after I had started my PhD research in Cambridge as one of R. A. Fisher’s last graduate students, and stimulated by my recent computing experience, I had begun programming the Cambridge EDSAC 2 computer to analyse numerically some population genetic models that had no obvious analytical solutions, and which included the simulation of stochastic effects. This turned out to be one of the first examples of such computer simulation, as was noted in a short editorial in Nature, and was another major jump in the appreciation of what could be achieved by electronic computers even at that time.

My next step was as a Post-Doctoral fellow at Stanford with Nobel Prize winner and extraordinary polymath, Joshua Lederberg. I later became a faculty member in his Genetics Department. With my computing background, unusual at that time even at Stanford, I was able to use the then available facilities for statistical analyses which could hardly have been done in any other way. That was still only arithmetic, but of a more complex nature, and hugely beneficial for the medically relevant work I was then doing. Initially I used the Stanford University’s central computing facility for various statistical analyses but soon transitioned to the excellent time sharing, terminal accessed facility conceived by Lederberg specifically for the Medical School and managed and developed by Ed Feigenbaum. It was not until I became Director of the Imperial Cancer Research Fund (ICRF) in 1979, after 9 years in Oxford, that I was able to set up, with my late wife Julia Bodmer, comparable computing facilities to those developed by Lederberg and Feigenbaum at Stanford in the mid- to late 1960s.

Lederberg was very keen on the use of the most up to date computing equipment, including the then novel computer driven printer whose lines were wriggly rather than smooth. When I submitted figures based on the printer for publication, the proofs came back with a presumably mistakenly included handwritten message saying,” What crazy person drew these wriggly figures, they will have to be re-drawn”. There is a good example of when a computer did not properly mimic human activity, something I often feel about current spell checkers!

Lederberg, not Feigenbaum as stated by Wooldridge, was the initiator of DENDRAL while the idea of expert systems was developed extensively with Ed Feigenbaum. The aim of DENDRAL was to predict chemical structures from their mass spectra using an expert knowledge base developed with the famous organic chemist Carl Djerassi, one of the world’s experts in the application of mass spectrum analysis for the determination of chemical structures. This was my first exposure to AI. (For more information on Joshua Lederberg see Bodmer and Ganesan [2] and the biography by Jan Sapp [6]).

At the ICRF, with this minimal background in computer developments and AI based on expert systems, I was intrigued by the possibility of their application to aid General Practioners in their diagnosis of cancer. This led to the appointment of John Fox to lead a laboratory for studying the applications of computing and knowledge-based systems to medical diagnosis and related areas. John had had post-doctoral experience working with Allen Newell and Herbert Simon and later came to Oxford to continue his work in the biomedical area as a professor in the department of engineering.

At the ICRF I also sought the advice of Donald Michie for our computer developments. Donald Michie was essentially the founder of AI in the UK, having been stimulated originally by his association with Alan Turing and the code breakers in Bletchley during the second world war. He was very seriously affected by Lighthill’s extraordinarily short sighted and dismissive 1973 report on AI.

## My Thoughts on AI (p. 174, etc., Refer to Page Numbers in Woolldridge [7])

My understanding of Machine Learning (p. 54) is that it can apply to both unsupervised and supervised analysis. Neural networks and their developments seem to be able to apply almost only to supervised machine learning, which is in many ways conceptually less challenging than unsupervised analysis. Classical statistical approaches, including, for example, clustering and phylogenetic estimation, initiated machine learning, while random forests now seem to be taking over. Supervised analysis using neural networks works very well, of course, for facial recognition, but it is unsupervised analysis that is needed for the identification of facial features for genetic analysis (see e.g., [3]).

The topical use of the example of virus detection, using an ‘Empirical Bayes’ approach, illustrates very clearly how for rare events there is a serious problem of the balance between false negatives and false positives. Depending on the consequences of finding a positive result, having more false than true positives can be a serious problem, and this is not unique to AI issues (pp. 155–157).

In spite of a very clear description of neural nets, I still find it difficult to understand why they work. It seems to be more of an art than a rationally established skill to get the best results from a neural net analysis. Perhaps it is my training as a statistician that makes it hard for me to appreciate how they might work, given that it still appears that nobody yet knows why they really work. Is that a job now for statisticians? (p. 174ff, see also [4])

Although using AI for the combined analysis of multiple sources of health-related data for individualised health guidance may be very useful (p. 280), the health information from wearable devices is still very limited. There is a continuing problem of balancing the amount of information needed for supervised analysis of health and medical issues with the needs for privacy of information.

The trolley problem of whether you should pull a lever to avoid killing 5 people rather than one illustrates the great difficulty, if not impossibility, of introducing morality into the control of driverless cars. I wonder, however, why there is no option for the trolley driver to kill her/himself, surely a likely possibility for a driverless car (pp. 247–253).

The effect of AI on the future of jobs is a much discussed question. There is surely no doubt, in spite of Wooldridge's comment, that tractors really did replace farmers (p.270)! Someone who once lived in my house told me that when he lived there, it was a farmhouse for a farm with ~ 30 + workers and now there were ~ 3–4. Thus, while the use of tractors replaced farmers, the industrial revolution created new types of jobs. The hope must be that AI will do the same, even if it is mostly leisure or intellectual activities that computers may never learn to displace!

## AI Needs to Confront Biology

Wooldrige's discussion of the sense of smell of coffee implies that we do not know much about such senses (p. 307), but the mechanisms by which these senses are detected are pretty well understood at the cellular and molecular levels. If ‘qualia’ refers to the experiential outcome of tasting coffee then that is a different and possibly quite individual matter, relating to the function of the mind rather than just the brain.

Thomas Nagel’s proposed test of consciousness (pp. 307–310) shows, in my opinion, a remarkable lack of understanding of the biology. Why stop at earthworms? What about yeast, then bacteria then viruses and even plants? Is not the main issue the lack of a central nervous system, namely a brain? Nagel’s reference to bats having a consciousness different than humans because they have senses, such as sonar, which humans do not have seems naive (p. 309). It is easy to conceive of how animals could deal with magnetic sensing, UV vision and ultrasound hearing using the same principles of sensing and communication with the brain as for visible light, sound, taste and smell. There is no reason to think that the impact of these senses in a bat on its brain will be any different than that of the usual human or any other animal senses.

Michael Wooldridge rightly criticises Hubert Dreyfus’ claim that computers could not deal with intuition (p. 311). The counter argument is that there is both inherited experience, namely instinct, which even humans have and which can be modelled, as well as intuition, which can be explained, at least in part, by accumulated and remembered experience.

Why should the detection of a decision 10 s before being ‘conscious’ of it be surprising (p. 315–316)? Think of the distinction between remembering a name and recalling it? You can spend much time trying to recall a name, and then, when you think about other things, suddenly you recall the name, which must have been stored in your memory. While you are consciously thinking of other things your brain has been at work searching for the name to be recalled. That begs the question of what consciousness is. Recalling in this way is, I assume, subconscious. What is the distinction, if any, between consciousness and awareness?

Michael Wooldridge argues that when a child ‘deliberates over which chocolate to choose from a selection’ the choice is careful, deliberate and purposeful (p. 319). Surely, however, there is likely to be at least some element of chance in the child’s choice of which chocolate to take. In general, such choices can often include a significant element of randomness. I know that is the case for myself! AI approaches in general seem to ignore the stochastic element of decisions and actions. Another similar situation involves the decision whether or not to go out with an umbrella (pp. 321–322). The argument about whether or not to take out the umbrella started with the belief that it might rain but turned into knowing whether it is or is not raining. The more the belief that it is going to rain increases, the more likely it is that one will take an umbrella. Here, probability matters.

## Could Computers Ever Evolve Without Human Intervention?

A biologist might have chosen the story of genetics and DNA as the apparent mystery of the mechanisms of inheritance that has been solved, in preference to the appreciation of the amount of energy produced by stars, solved by nuclear fusion (p. 305). Will the mystery of what AI with computers of unbelievable power may eventually achieve be solved in the same way? Will computers ever exceed human intelligence and then take over the world?

There is no life without self-reproduction. Any replicating system in which heritable variants with differing replicative potentials can arise is subject to a Darwinian process of evolution by natural selection. Darwinian evolution ultimately underlies all aspects of understanding biology and that must include the workings of the brain and its relation to the mind. I remember a meeting with Joshua Lederberg, Marvin Minsky and John McCarthy in which they tried to persuade me that computers will evolve to succeed humans. But that cannot happen unless computers learn how to replicate themselves completely without human intervention and do so while continually improving their replicative potential.

The term ‘self-replicate’ occurs only once in the book (p. 255) under the heading of ‘Recursive Self-Improvement’. While that could cause problems, it is not replication. Neither self-reproduction nor replication are in the index, and yet without these, computers can never be an existential threat to humans, unless they unintentially initiate a nuclear explosion that kills us all.

What could be very dangerous is the genetic manipulation of a virus or other infectious organism to become highly virulent and easily spread, say a particularly nasty version of corona virus SARS-2, ‘Asian’ flu or anthrax. The danger lies in the ability to reproduce. That is why Joshua Lederberg spent a great deal of effort arguing against biological warfare, rightly considering it far more dangerous than chemical warfare.

What are the ways in which computers could directly become dangerous without self- replication? For example, drones under remote computer control can become autonomous lethal weapons. Can they be made to be “moral”? How can or should one control their use and development by legislation at an international level (pp. 282–287)?

What is ethical AI? Does it exist and, if so, how could it be defined? Can there be ethical mathematics? Michael Wooldridge argues that we cannot legislate to limit the general use of AI any more than one could the use of mathematics (pp. 253–262).

I think the worst and most objectional development of AI would be to be able to read someone else’s mind.

## Where Next for the Mind/Brain Relationship?

Using a computer-based approach on its own, without understanding more about the function of the brain at the cellular and molecular level, is surely not the answer for understanding the mind/brain relationship. There is a fundamental problem in the computer-based approach since, even if we know all the possible inputs and outputs of a complex machine, that will never be enough on its own to work out how the machine works. We must bring together the biological and evolutionary knowledge about the brain at the cellular and molecular level with behavioural and cognitive studies.

That is where I think genetics can play a huge role. Thus, I have been involved in proposals aimed at identifying specific genetic variants that could account for some measurable features of, for example, extremely high mathematical or musical ability. Then, knowing the molecular and cellular function of such a variant might be a route to finding out something about the molecular basis for such extreme abilities, and so to connecting some aspects of the mind with specific brain functions.

## What Makes Us Human?

This is a question often asked and not easily answered, but obviously relevant to the question of whether computers will ever match human intelligence and become ‘Conscious Machines’.

Non-human animals must surely have elements of the mind/brain relationship we often consider only in a specifically human context. Why should we assume that only humans can create an imagined world for themselves? Beyond this then, it seems most likely that it is ultimately the level of cognitive ability that distinguishes humans from other animals.

I do not believe brain size is the key reason, though it is undoubtedly important and may be a requirement. Complexity or efficiency of function must also be a factor. Perhaps complexity of function came first and then that led to selection for increased brain size. Dunbar’s argument for a larger group size for humans than that for the higher primates is convincing and receives support from others (pp. 317–319). I would suggest that the difference has more to do with the notion that, with increased cognitive ability (which must not be confounded with consciousness) and better communication between individuals, altruism extended beyond immediate relatives. Altruism amongst genetically closely related individuals was the limit proposed originally by Haldane and Fisher and famously enlarged on by William Hamilton [5]. Larger groups became advantageous when internally interconnected by communication and altruism, and that may also be the basis for the early evolution of religions (see [1]).
